# The seasonality experiment: Investigating how seasons affect the burning conditions of cremations

**DOI:** 10.1371/journal.pone.0327478

**Published:** 2025-07-09

**Authors:** Elisavet Stamataki, Guy De Mulder, Rosalie Hermans, Martine Vercauteren, Christophe Snoeck

**Affiliations:** 1 Archaeology, Environmental Changes, and Geo-Chemistry Research Group, Vrije Universiteit Brussel, Brussels, Belgium; 2 Research Unit, Anthropology and Human Genetics, Department of Biology of Organisms and Ecology, Université Libre de Bruxelles, Brussels, Belgium; 3 Department of Archaeology, Ghent University, Ghent, Belgium; Institute for Anthropological Research, CROATIA

## Abstract

This study investigates the influence of seasons and weather conditions on cremation processes, using experimental archaeology and advanced analytical techniques. Four outdoor cremation experiments were conducted across different seasons in Greece, during which the four fleshed legs (front and hind) of the same domestic pig (*Sus scrofa*) were burned. The study highlights the effects of variables such as temperature, dryness of fuelwood, atmospheric pressure, and precipitation on the structural and chemical composition of burned bones. Results demonstrate a strong correlation between burning conditions and isotopic (δ^13^C, δ^18^O) as well as infrared indices (Infrared Splitting Factor (IRSF), Carbonyl-to-Carbonate ratio (C/C)), which are temperature-related. Comparisons with archaeological data from Belgium reveal potential seasonal patterns in past cremation practices. The findings underscore the need for expanded experimental research in various geographical areas with different altitudes and weather conditions to further investigate how the location in which burning was performed in combination with weather conditions affected the cremation settings and therefore past funerary practices.

## Introduction

Burned human bones are commonly found in archaeological contexts since cremation was a common funerary practice in past societies [1]. The complexity of studying cremated bones lies in the high state of fragmentation of the remains and the structural and chemical alterations caused by the high temperatures (> 700 °C) reached during the burning process [2–6]. Analysing and interpreting cremated human remains involves a wide range of themes such as demographic data, evidence of pathological lesions [e.g., 7–14], diet and mobility [e.g., 15–22], as well as topics about funerary practices and pyrotechnology [e.g., 23–28].

Regarding funerary practices, developments in carbon and oxygen isotope analysis, radiocarbon dating [16,28–42] and Fourier Transform Infrared Spectroscopy in the Attenuated Total Reflectance mode (FTIR-ATR) [25,28,43–51] have provided new insights into the heat alterations of burned bones. These advancements have highlighted the increasing importance of analysing cremated remains to explore various aspects of pyre technology (such as temperature, pyre size, ventilation conditions, etc.) and body management in past societies where cremation was the main funerary practice [26–28,38,52].

In combination with analytical techniques, numerous laboratory and outdoor burning experiments have been conducted to investigate the effect of fire on the human body and the thermal decomposition of skeletal remains [e.g., 8,42,53–62]. Therefore, experimental archaeology through the reconstruction of funerary pyres contributes significantly to increasing our knowledge of how thermal alterations of burned bones are linked to different burning conditions [58–63].

While the study of experimental and archaeological cremated remains provides valuable insights into burning conditions and their relation to funerary rites, there is limited archaeological evidence about the season in which cremation was performed. The lack of written sources from the Metal Ages and the limited written sources from the Roman period makes it extremely difficult to approach questions related to the season and under which weather conditions cremation was performed. This creates a gap in our understanding of funerary practices from chronological periods when cremation was practiced, whether as a dominant or coexisting rite. Understanding the season of cremation is important, as it can shed light on the decision-making processes of living individuals concerning funerary practices influenced by societal rituals, wood availability, and weather constraints. Therefore, it is necessary to turn to other types of evidence, such as experimental archaeology and/or ethnographic information, to further investigate the season and the weather conditions during combustion.

McKinley [54,55,63] assumes that the climate would have significantly influenced the timing of cremations, especially in areas where the weather is often rainy and windy. Since the weather conditions could not be controlled, McKinley [54,63] believes that it might have been necessary to wait for a relatively dry day to perform a cremation and that in certain seasons and areas, this waiting period could have stretched to several weeks. Based on ethnographic evidence from India, the Kol people, when it was too rainy to perform cremation, used to first bury the body for a while following specific rites and then cremate it in a ceremony of three stages [64]. It is expected that funerary pyres would not ignite during heavy rain or if the wood was humid. Strong, veering winds could cause a heterogeneous burning and possibly the collapse of the pyre, with the unwanted slipping of the body, making necessary the presence of someone to manage the funerary pyre and the human body during the burning process. Additionally, heavy, persistent rain could have extinguished the pyre causing also an uneven burning [54,55,63]. This happened during the cremation of the astrologer Asceletario, who was executed by Emperor Domitian (ca. 81–96 CE). During his cremation, a sudden storm extinguished the funerary pyre leaving his body half-burnt (Suetonius, Domitianus 15, as cited in Noy 2000 [65]). In another case, the cremation of Sulla was delayed for hours because the day was cloudy. During the cremation, a strong wind helped and speeded up the burning process while heavy rain started falling right after the bone collection (Plutarch, Sulla 38, as cited in Noy 2000 [65]). It seems, that uncontrolled factors, such as the season and the weather conditions in which cremation occurred did not only influence the timing of the cremation but the whole burning environment.

This study aims to combine experimental archaeology and state-of-the-art analytical techniques to investigate how alterations in the structural and chemical composition of burned bones can provide information regarding the season and the weather conditions in which cremation was performed. Exploring how weather conditions affect the burning process and therefore the structural and chemical composition of burned bones is important for understanding in which season cremation was performed in past societies.

## Materials and methods

### Design of the experiment

To investigate the effect of seasons and weather conditions on the structural and chemical composition of burned bones, the four fleshed legs (front and hind) from the same individual domestic pig (*Sus scrofa*) were obtained from a local Greek butchery and burnt in a series of outdoor experiments in different seasons starting in summer 2022 (summer 2022, autumn 2022, winter 2023, spring 2023) (Table 1). This approach ensured isotopic consistency across samples, as intra-individual variability is generally minimal. For all experiments, the distal portions of the limbs—specifically the radius and ulna for the front legs, and tibia and fibula for the hind legs—were selected to standardise body part selection and minimise variability in thermal exposure due to anatomical differences. This allowed for more direct comparison of burning behaviour and bone alteration across seasons.

**Table 1:** Climatic information for the month and the day of the experiments and average recorded temperatures of the pyre and leg.

Season	Element	Day Temperature (°C)	Monthly Mean Temperature (°C)	Monthly Max Temperature (°C)	Monthly Min Temperature (°C)	Monthly Precipitation height (mm)	Monthly precipitation anomaly (mm)	Humidity of the day (%)	Wind speed (km/h)	Wind direction	Atmospheric pressure (hPa)	Average Pyre T (°C)	Average Leg T (°C)
**Winter** **2023**	Hind	10	9.4	14.5	5.1	62	10	70	5	NW	1013	613	472
**Spring** **2023**	Front	16	18.6	22.5	11.9	14	24	75	12	N	1018	647	524
**Summer** **2022**	Hind	26	28	28	19.8	27	10	30	24	SW	1019	595	462
**Autumn** **2022**	Front	18	17.9	17.9	12.7	9	−6	40	10	NW	1022	625	483

Although anatomical differences between front and hind limbs in pigs may affect burning due to variations in muscle and fat composition, no significant difference in muscle-to-fat ratio was observed in the specific parts used in this study. Pigs were selected as experimental models due to their anatomical and soft tissue similarities to humans, particularly in the structure of skin, fat, and muscle layers, which make them suitable analogues for experimental research [66]. To ensure comparability across experiments performed in different seasons, the legs were stored frozen at −4 °C until the time of each burning experiment. Prior to burning, they were removed from the freezer and left to thaw and reach ambient temperature for approximately 24 hours.

All the experimental burnings were conducted in the same location at the village of Martino (38.6253° N, 23.2182° E) in central Greece. The village of Martino is located 5.86 km from the sea at an altitude of 256 m. The experiments were carried out on private land owned by the first author. No live animals were used, and the remains were sourced post-mortem from animals slaughtered for commercial purposes, in accordance with national regulations. As the site is privately owned and the materials used were legally obtained animal remains, no permits or field site access approvals were required.

Regarding the burning settings, the origin of the fuelwood, the type and amount of wood (olive tree trunks/ *Olea europaea*), the pyre structure (rectangular shape and layers of logs placed perpendicular to the one before), the size of the pyre (0.5 m x 0.5 m x 0.5 m), the position of the leg on the top of the pyre, and the duration of burning (4 hours) were the same in each experiment (Table 1). Based on the volume and density of olive wood, the estimated weight of fuelwood used per experiment was approximately 110–115 kg. The fires were always ignited from the bottom of each pyre using a blow torch with butane cartridge.

A difference was observed in the dryness of fuelwood used for the different experiments. The olive trunks were fully dry for the experiments that were carried out in summer 2022 and autumn 2022, but they were partially humid for the experiments in winter 2023 and spring 2023, because they were stored outside and were only partially covered and protected from rainfall. The temperature was recorded every 15 minutes on the four sides of the pyre (N, E, S, and W sides) and the leg using a PCE-890U infrared thermometer [67]. In all cases, the burned pig bones were carefully collected the next morning and, in total, 26 samples were analysed for this study (n = 6 summer 2022; n = 8 autumn 2022; n = 7 winter 2023; n = 5 spring 2023) [67].

According to the climatic report of the Hellenic National Meteorological Service (HNMS) [68] for the region of the experiments (Table 1), July 2022 was a warm but rainy month. The monthly temperature ranged from 19.8 °C to 31.9 °C with a mean temperature of 28 °C. The monthly precipitation height was 27 mm with a monthly anomaly of 10 mm (Monthly Anomaly = Observed Precipitation − Long-term Average Precipitation). According to HNMS, a positive anomaly indicates more precipitation than the recorded long-term average, representing wetter conditions. A negative anomaly indicates drier weather conditions. At the time of the experiment, the ambient temperature was 26 °C, the humidity was 30%, the wind was 24 km/h with SW direction, and the atmospheric pressure was 1019 hPa.

October 2022 was a warm and relatively dry month for the area in which the experiment was performed with temperatures higher than normal [68]. The monthly temperature ranged from 12.7 °C to 23.1 °C with a mean temperature of 17.9 °C and the monthly precipitation height was 9 mm with a monthly anomaly of −6 mm. At the time of the experiment, the ambient temperature was 18 °C, the humidity was 40%, the wind was 10 km/h with NW direction, and the atmospheric pressure was 1022 hPa (Table 1).

January 2023 was also a warm month, and the monthly temperature was ranging from 5.1 °C to 14.5 °C with a mean temperature of 9.4 °C [68]. The mean precipitation height (62 mm) was higher than normal for the region. The monthly precipitation anomaly was 10 mm and large amounts of snow were recorded over the tops of the mountains (Parnassos and Giona) during the last ten days of the month. At the time of the experiment, the ambient temperature was 10 °C, the humidity was 70%, the wind was 5 km/h with NW direction, and the atmospheric pressure was 1013 hPa (Table 1).

May 2023 was a relatively cold month for the region [68]. The monthly temperature was ranging from 11.9 °C to 22.5 °C with a mean temperature of 18.6 °C. The mean precipitation value was higher than normal with a monthly precipitation height of 14 mm and a monthly anomaly of 24 mm. At the time of the experiment, the ambient temperature was 16 °C, the humidity was 75%, the wind had speed 12 km/h and N direction, and the atmospheric pressure was 1018 hPa (Table 1).

## Methods

### Pre-treatment of calcined bone fragments

Following the recovery of the bones after the burning experiment, approximately 200 mg of burned bone fragments were pre-treated for carbon and oxygen isotope and FTIR-ATR analyses at the Archaeology, Environmental, and Geochemistry (AMGC) research unit at the Vrije Universiteit Brussel (VUB), Belgium, to eliminate secondary carbonates and post-burial contamination. Mechanical cleaning was performed via drilling as described by Stamataki et al. [28], followed by chemical pretreatment following Snoeck et al. [69]. Samples underwent six 10-minute ultrasonic baths in MilliQ water, one in 1 M acetic acid (CH_3_COOH), and six more in MilliQ water before drying overnight at 50 °C and being crushed.

### Carbon and oxygen isotope analyses of bone apatite carbonates

For carbon and oxygen isotope analysis of bone apatite carbonates, 30 mg of bone powder (15 mg per duplicate) was processed following Stamataki et al. [28]. The powder was placed in sealed glass tubes (exetainer® from Labco Limiter), flushed with helium to remove oxygen, and treated with phosphoric acid to extract CO_2_. The extracted CO_2_ was analysed using a Nu Perspective IRMS with a Nu GasPrep bench at the Vrije Universiteit Brussel. Isotope ratios are expressed as δ units, showing deviations from a specific standard value [70–71]. The results are reported as per mil (‰) deviation from the VPDB reference standard. International standards (IA-R022, IAEA-603, and IAEA-CO8) were used to calibrate the isotopic values and the analytical precision was better than ± 0.30‰ (δ^13^C) and ± 0.40‰ (δ^18^O) based on repeated measurements of in-house cremated bone standard CBA (n = 15; see De Winter et al. [72]).

### Fourier transform infrared spectroscopy in attenuated total reflectance mode (FTIR-ATR)

FTIR-ATR analysis was conducted on 6 mg of sieved bone powder (25−50 μm fraction) following Stamataki et al. [28], as this fraction provides reliable and reproducible results [73]. Each sample was analysed in triplicates (~2 mg per measurement), and the reported indices are averages of the three measurements. Analyses were performed at AMGC-VUB using a Bruker Vertex 70v FTIR spectrometer (4000–400 cm ⁻ ¹ range, 32 scans, 4 cm ⁻ ¹ resolution) under vacuum. The crystal plate and anvil were cleaned with isopropanol after each measurement. Spectra were processed with OPUS 7.5 software and all the indices were calculated after the baseline correction (see SI of Stamataki et al., [28]).

### Statistical tests

IBM SPSS Statistics version 29.0.1.0 was used to perform univariate and multivariate statistical tests to assess the significance of variations in measured attributes across the examined seasons. Before any statistical comparison, the Gaussian distribution was examined using the Kolmogorov-Smirnov test. As infrared and isotope variables followed a normal distribution, parametric tests including MANOVA and Independent Samples *t*-test were applied to evaluate differences among seasons. Statistical significance was set at p ≤ 0.05.

To explore seasonal patterns in the archaeological samples, z-score standardised δ¹³C and δ¹⁸O values were compared to seasonal reference centroids using Euclidean distance. A chi-square test of independence assessed the relationship between assigned season and archaeological period. Full methodological details are provided in S1 Text File, and the dataset used is available in S2 Excel Table.

**Results and discussion**

The following section presents the results of the experimental cremations, beginning with observations of burning conditions and temperature variations recorded during each seasonal experiment. These are followed by the analytical outcomes of infrared spectroscopy and carbon and oxygen isotope analyses. Figures and tables are provided to illustrate temperature trends, weather conditions, and the structural and compositional changes in samples burned across different seasons. The “Average pyre” and “Average leg” values in Table 1 represent the mean of all these recorded measurements across the burning period.

**Fig 1:**
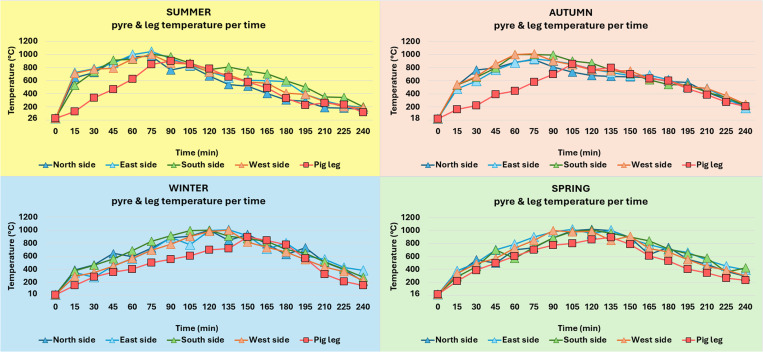
Recorded pyre and leg temperatures per season. The temperature was recorded every 15 minutes for the four sides of the pyre (N, E, S, and W sides) and the leg.

Comparing the burning process among the different seasons, it was observed that in summer, the moderate speed of the wind (24 km/h) on the day of the experiment, in combination with the high temperature of the day (26 °C), and the use of fully dried fuelwood led the temperature of the leg to reach the highest recorded temperature more quickly- within just 90 minutes after the ignition of the pyre (Fig 1). It was also noted that the temperature rose faster in summer and autumn, when the olive trunks were fully dried, compared to winter and spring, when the fuelwood was partially humid (Fig 1) [67].

**Fig 2:**
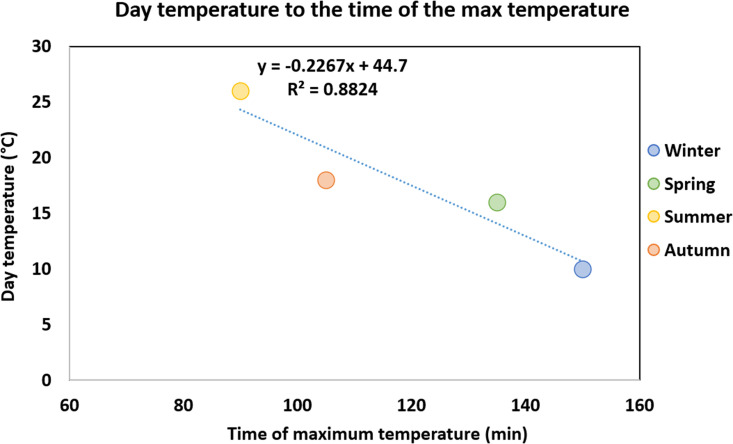
Scatter plot of the temperature of the day in which each experiment was performed and the time it took for each leg to reach the maximum temperature with regression line (Pearson correlation).

Furthermore, the temperature of the day on which each experiment was performed strongly correlates with the time it takes for each leg to reach maximum temperature (r = 0.94, 95% CI: 0.75–0.95, p < 0.01). The coefficient of determination (R²) indicates that 88% of the variation in time to maximum temperature is explained by the daily temperature. It seems that the higher the temperature of the day the shorter the time needed for each leg to reach its maximum temperature (Fig 2) [67]. However, the highest mean pyre (647 °C) and leg (524 °C) temperatures were recorded in spring (Table 1), even though humidity was high (75%), the wood was partially moist, and the maximum temperature was achieved 135 minutes after the ignition of the pyre (Fig 1). Surprisingly, the lowest mean pyre (595 °C) and leg (462 °C) temperatures were measured in summer and not in winter as was expected based on the weather conditions and the state of the fuelwood (Table 1). Nevertheless, in the summer experiment, the temperature reached the highest recorded temperature of all four experiments, 1040 °C, but only for a few minutes [67].

**Fig 3:**
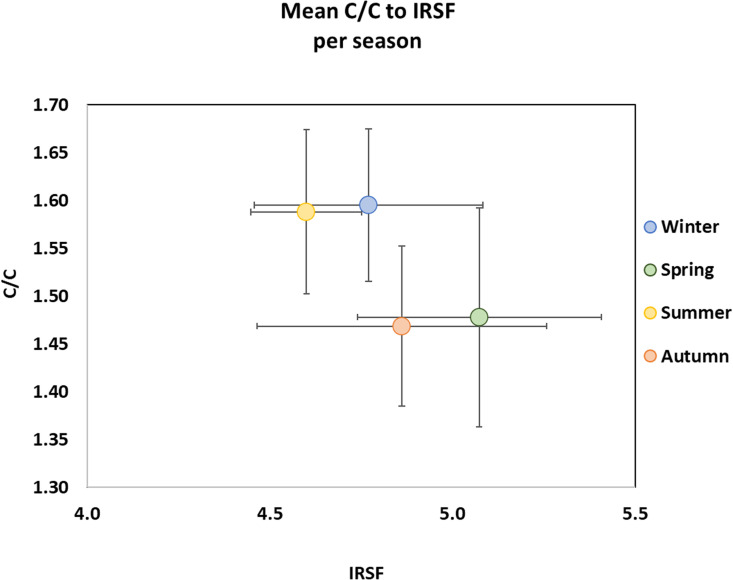
C/C to IRSF of all the burned skeletal elements per season (mean ± 1 SD).

Experimental research for forensic and archaeological sciences has proved that the infrared splitting factor (IRSF) to carbonyl-to-carbonate ratios (C/C) are temperature-related and they can be used as indicators for distinguishing low intensity (< 600 °C) from high intensity (> 600 °C) burnings [25,43,50,74,75]. As expected, based on the recorded mean pyre and leg temperatures, the burned pig bones from autumn and spring present higher mean IRSF and lower mean C/C ratios compared to the experimental data from summer and winter, proving that the mean burning temperature was higher in autumn and spring with the burned bones from the spring to present the highest mean temperature and the highest mean IRSF value (Fig 3, Table 2) [67]. It seems that the average temperature during combustion is more important than the maximum temperature, as it influences IRSF and C/C ratios. The difference in IRSF and C/C ratios between summer and winter, and autumn and spring are statistically significant (Independent *t*-tes*t* for IRSF: (t)24 = −1.994, p = 0.05, Cohen’s d = −0.782, 95% confidence interval [−1.574, 0.025]; for C/C: (t)24 = 3.564, p = 0.002, Cohen’s d = 1.389, 95% confidence interval [0.523, 2.250]). At the beginning of the experiment, it was expected that the mean pyre and leg temperatures would be higher in summer compared to the other seasons considering that fully dried wood was used for the experimental pyre, the day temperature was relatively high (26 °C), and a moderate wind (24 km/h) facilitated the burning process. However, those expectations were not entirely met, as the mean pyre and leg temperatures in summer were lower than anticipated.

**Table 2:** Mean values and standard deviations (SD) for infrared indices and δ^13^C and δ^18^O values of all the burned skeletal elements per season.

Season	IRSF	SD	C/C	SD	δ^13^C (‰)	SD	δ^18^O (‰)	SD
**Winter 2023**	4.8	0.31	1.60	0.08	−24.1	2.3	−17.3	1.3
**Spring 2023**	5.1	0.33	1.48	0.11	−23.3	3.0	−16.0	1.3
**Summer 2022**	4.6	0.15	1.59	0.09	−23.3	0.8	−15.6	1.0
**Autumn 2022**	4.9	0.40	1.47	0.08	−22.7	1.8	−15.3	1.8

**Fig 4:**
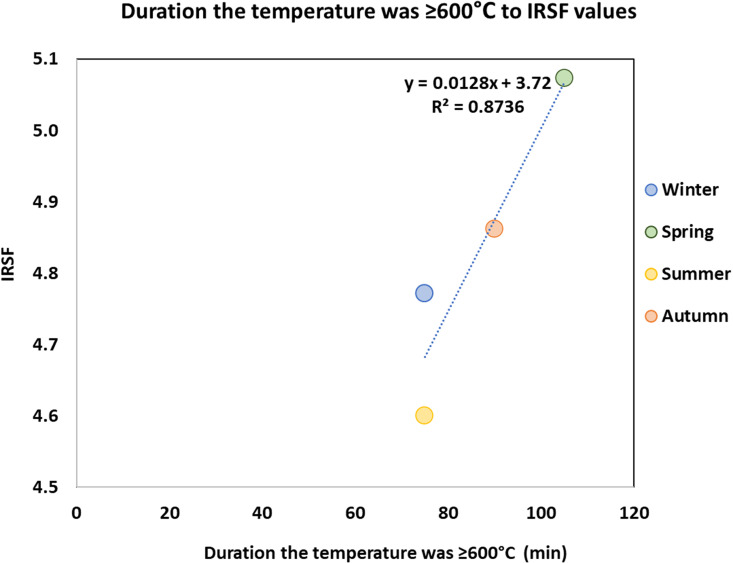
Scatter plot between the duration the temperature was equal or higher than 600°C and mean IRSF (± 1 SD) values of burned bones per season with regression line (Pearson correlation).

A strong linear correlation (R^2 ^= 0.87) was also noticed between the duration for which the temperature of the legs was equal or higher than 600 °C and the mean IRSF values of the burned bones for each season (Fig 4) [67]. The Pearson correlation between the two variables is statistically significant (p < 0.02) and the 95% confidence interval for the Pearson correlation coefficient ranged from 0.77 to 0.94, indicating the strength of this relationship. It seems that IRSF is not only temperature-related, but it is also linked to the duration of high intensity burning. The season in which each experiment was performed did not seem to influence the duration of high intensity burning as indicated by the same duration (75 minutes) in which the leg temperatures were equal or higher than 600 °C in summer and winter.

Differences were not observed regarding the state of fragmentation of the studied burned skeletal remains among the different seasons. The collection of the burned bones took place approximately 24 hours after each experiment. According to the research of Waterhouse [58], an increased fragmentation has been recorded when the temperature fluctuates around 0 °C on the day of collection. It was also shown that rainy weather conditions on the day of the recovery caused increased fragmentation and when there is a delay in the collection of the skeletal remains both fluctuating temperatures and freezing conditions have also a large impact on the state of fragmentation. In the seasonality experiment, even in winter, the lowest temperature recorded on the day of the collection was 8 °C. Furthermore, the humidity was high during the experiments in winter (70%) and spring (75%), but rainy weather conditions on the day of the experiment or the day of the recovery were not observed in any experiment. As a result, the lack of freezing temperatures or rainy weather conditions, in combination with the fast collection of the skeletal remains could explain the lack of extreme cracking and fragmentation of the burned bones in this study.

**Fig 5:**
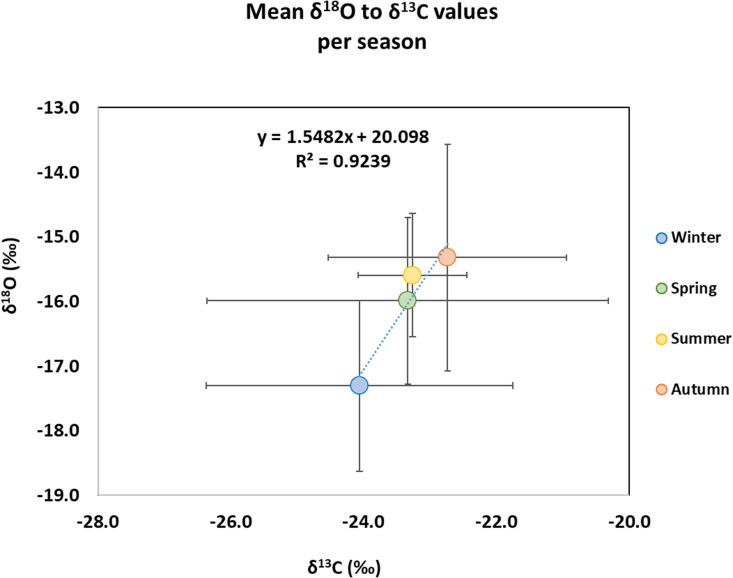
δ^18^O to δ^13^C values of all the burned skeletal elements per season (mean ± 1 SD).

Regarding the carbon and oxygen isotope results (Fig 5, Table 2) [67], a large variability can be observed in δ^13^C values of all the skeletal elements in spring and winter, with values ranging from −27.5‰ to −19.7‰ and from −27.1‰ to −20.8‰, respectively. Their variability in δ^18^O values is smaller (spring: from −17.4‰ to −13.9‰; winter: from −19.1‰ to −15.0‰). Autumn shows smaller variability in δ^13^C values (from −24.7‰ to −19.3‰) compared to spring and winter, but the largest variability in δ^18^O values (from −17.7‰ to-12.8‰) among all the seasons. Summer has the lowest variability in both δ^13^C (from −24.8‰ to −22.5‰) and δ^18^O values (from −16.7‰ to −16.7‰). The burned skeletal remains of the winter experiment present the lowest mean δ^13^C and δ^18^O values and autumn shows the highest among all the seasons (Fig 5). The difference in δ^18^O values between winter and autumn is statistically significant (Independent *t*-test: *t*(13) = −2.432, p = 0.03, Cohen’s d = −1.259, 95% confidence interval [−2.361, −0.117]) but in δ^13^C values is not (Independent *t*-test: *t*(13) = −1.251, p = 0.23, Cohen’s d = −0.647, 95% confidence interval [−1.679, 0.408]). A MANOVA test further proved the difference in δ^13^C and δ^18^O values between winter and the other three seasons (p = 0.002, Pillai’s Trace value = 0.41677). Furthermore, a strong linear correlation (R^2 ^= 0.92) was observed between the mean δ^13^C and δ^18^O values of all the burned skeletal elements (Fig 5). This correlation is statistically significant (p < 0.01) and the 95% confidence interval for the Pearson correlation coefficient ranged from 0.83 to 0.97, further supporting the strength of this relationship.

**Fig 6:**
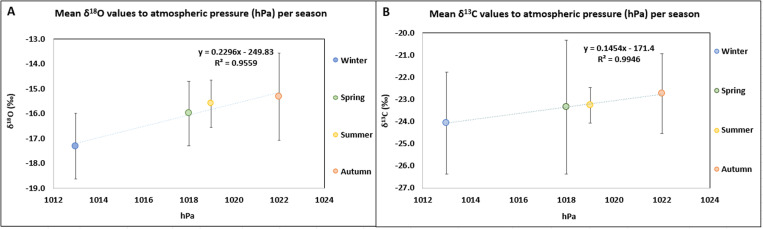
A) Scatter plot between atmospheric pressure (hPa) and mean δ^18^O values (± 1 SD) per season with regression line (Pearson correlation: R^2 ^= 0.96; 95% confidence interval ranged from 0.90 to 0.98). B) Scatter plot between atmospheric pressure (hPa) and mean δ^13^C values (± 1 SD) per season with regression line (Pearson correlation: R^2 ^= 0.99; 95% confidence interval ranged from 0.98 to 0.99).

**Fig 7:**
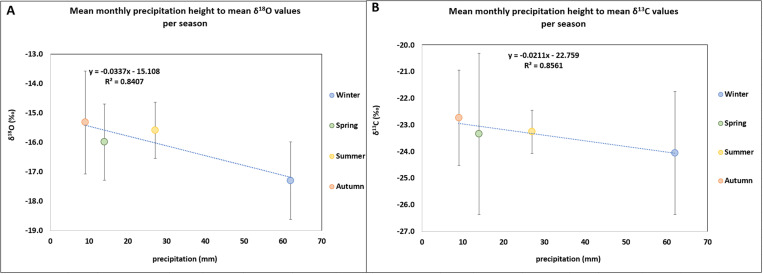
A) Scatter plot between mean monthly precipitation height (mm) of the region and mean δ^18^O values (± 1 SD) per season with regression line (Pearson correlation: R^2 ^= 0.84; 95% confidence interval ranged from 0.67 to 0.93). B) Scatter plot between mean monthly precipitation height (mm) of the region and mean δ^13^C values (± 1 SD) per season with regression line (Pearson correlation: R^2 ^= 0.86; 95% confidence interval ranged from 0.70 to 0.93).

The atmospheric pressure (hPa) of the day each experiment was performed strongly affected the mean δ^13^C and δ^18^O values (Figs 6A and 6B): the lower the atmospheric pressure, the lower the mean δ^13^C and δ^18^O values. These mean δ^13^C and δ^18^O values were also affected by the mean monthly precipitation height of the region, but to a lesser extent (Figs 7A and 7B): the higher the mean monthly precipitation, the lower the mean δ^13^C and δ^18^O values of the burned bones. These relationship could probably explain the difference in mean δ^18^O values between the experiment that was conducted in autumn, in a day with high atmospheric pressure (1022 hPa) and with low mean monthly precipitation height (9 mm with monthly anomaly −6 mm) with the experiment that was performed in winter, in a day with low air pressure (1013 hPa) and high mean precipitation height (62 mm with monthly anomaly 10 mm). From the beginning of the seasonality experiment, it was expected that humidity would also affect the δ^18^O values among the different seasons but a relationship between the humidity on the day of the experiment and the δ^18^O values was not observed, as it was noticed between the δ^18^O values and the monthly precipitation height.

**Fig 8:**
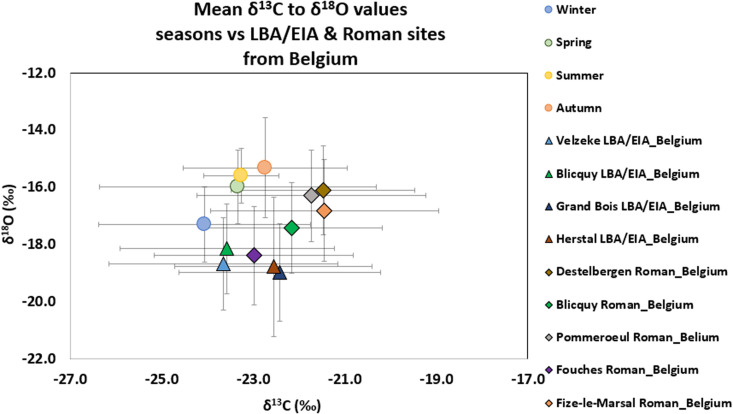
Mean δ^18^O to δ^13^C values (± 1 SD) of all the burned skeletal elements per season (this study) and the archaeological cremated bones from the LBA/EIA [28] and Roman period [77] from Belgium.

To assess whether it is possible to determine the season in which cremation was performed in past societies, the mean δ^13^C and δ^18^O values from the experimental data were compared with the only available archaeological δ^13^C and δ^18^O data from the Late Bronze Age (LBA)/ Early Iron Age (EIA) [28] and Gallo-Roman cremations [76–77] from Belgium (Fig 8, Table 3). In Stamataki et al. (76–77), it was shown that the Gallo-Roman cemeteries present more enriched δ^13^C and δ^18^O values compared to the LBA/EIA cemeteries, except for the cemetery of Fouches. This suggests that the Gallo-Roman cremations likely had better oxygen availability during combustion, which could be related to factors such as the pyre structure (i.e., lower oxygen to fuel ratio), the pyre’s location in the environment (e.g., on hill vs in a pit), the methods used to extinguish the pyre after burning (e.g., natural cool down vs use of water/wine to extinguish the pyre), and the season and weather conditions during cremation (e.g., cremations performed in months with low atmospheric pressure and high precipitation, such as winter, vs those with high atmospheric pressure and low precipitation, such as summer).

**Table 3:** Mean values and standard deviations (SD) for infrared indices and δ^13^C and δ^18^O values of all the burned skeletal elements from the LBA/EIA (* [28]) and Roman sites (** [77]) from Belgium.

	Mean Carbon (δ^13^C ‰)	SD	Mean Oxygen (δ^18^O ‰)	SD
Velzeke LBA/EIA*	−23.6	2.5	−18.7	1.6
Blicquy LBA/EIA*	−23.6	2.3	−18.2	1.6
Grand Bois LBA/EIA*	−22.4	2.2	−19.0	1.7
Herstal LBA/EIA*	−22.6	2.2	−18.8	2.4
Destelbergen Roman**	−21.5	2.0	−16.1	1.6
Blicquy Roman**	−22.2	2.0	−17.4	1.6
Pommeroeul Roman**	−21.7	2.5	−16.3	1.6
Fouches Roman**	−23.0	2.2	−18.4	1.7
Fize-le-Marsal Roman**	−21.4	2.5	−16.8	1.8

The comparison between the mean δ^13^C and δ^18^O values of the experimental and archaeological samples demonstrates that the Gallo-Roman cremated remains exhibit high variability in mean δ¹³C and δ¹⁸O values, spanning a wide range across multiple seasons (winter, spring, summer, and autumn), suggesting that cremations likely occurred throughout the year. In contrast, the LBA/EIA cremated remains show lower variability in mean δ¹³C and δ¹⁸O values compared to the Gallo-Roman samples, clustering around the range observed in the winter experimental data (Fig 8, Table 3). To further support this observation, the Euclidean distance between each archaeological sample and the mean values (δ13C and δ18O) of every season was calculated, and each sample was assigned to the closest season (winter, autumn, spring, summer) (see S1 Text for methodology and S2 Excel Table). Additionally, a chi-square test of independence with Yates’ continuity correction, using all the infrared and isotopic data, was conducted to examine the relationship between the LBA/EIA and Roman period and the distribution across the different seasons. The chi-square test indicates that the distribution of seasons between the LBA/EIA and Roman period is significantly different (χ^2 ^= 63.531, p < 0.00).

It is acknowledged that the seasonality experiment was conducted in modern-day Greece, while the archaeological data are derived from prehistoric and Roman-period Belgium. This introduces potential bias due to differences in climate, fuel types, and local environmental conditions. Furthermore, the present study included only one experimental cremation per season, and as such, these comparisons should be interpreted as tentative hypotheses, not definitive conclusions. Repeated experiments under a wider range of environmental and burning conditions are necessary to further explore how burning locations and weather conditions affect cremation settings.

Within these limitations, exploratory interpretations can be considered regarding why the LBA/EIA cremations were likely performed in winter or under winter-like weather conditions. One explanation may involve higher mortality rates during winter, a season often associated with increased illness due to harsher weather, food scarcity, and lower nutritional quality, which can weaken immune systems and increase vulnerability to diseases [78–79]. This seasonal peak in mortality might result in more cremations being performed during winter months.

Practical reasons may also explain why Belgian LBA/EIA cremations were likely performed in winter. Cremation requires significant amounts of fuelwood (200 to 600 kilograms) based on ethnographic information from Hindu cremations [80–81]. In agricultural societies, wood may have been more readily available for funerary practices during the winter, having been stockpiled for both heating and cremation purposes. Additionally, cremation is a time-consuming process that likely requires community involvement, so it would make sense to perform these rites in winter when there were fewer demands on agricultural activities such as planting and harvesting [82]. During periods of harvest or planting, people were likely more dispersed, working in the fields or tending livestock, whereas in winter, it was probably easier for people to stay close to their settlements and to organise and participate in funerary rituals. By contrast, in the Roman period, cremation practices may have been performed across a wider range of seasons indicating probably more organised funerary practices and/or the availability of fuelwood year-round.

Furthermore, historical written sources also offer valuable context regarding the timing of cremations. For example, Wulfstan’s 9th-century account of the Aesti tribe (from the southeastern coast of the Baltic Sea) describes a prolonged interval between death and cremation, during which the body was kept cool to prevent decomposition and elaborate rituals such as feasting and gambling took place [83]. While this does not directly indicate a seasonal preference, it suggests that cremations could be delayed intentionally—potentially leading to seasonal patterns, especially in colder climates where natural refrigeration made long delays more feasible. Although this text originates from the early medieval period and a different cultural context, it highlights how environmental conditions and cultural practices could interact to shape the timing of cremation.

In this light, the winter cremation pattern observed in LBA/EIA Belgium could also have been influenced by a combination of environmental constraints and cultural choices. While the evidence does not point to a deliberate preference for winter cremation, practical and environmental considerations—such as ease of body preservation and fuel availability—could have contributed to the concentration of cremations during colder months. Incorporating such perspectives underscores the complexity of interpreting seasonality in cremation practices and supports the need for multidisciplinary approaches that combine archaeological, environmental, and textual evidence.

In conclusion, this study offers preliminary evidence that carbon and oxygen isotope analysis on bioapatite can be used for investigating the season in which cremation was performed, as both δ^13^C and δ^18^O values are affected by the atmospheric pressure and precipitation. However, more experimental research in different geographical areas with different altitudes and weather conditions is important for further investigating how the location in which burning was performed in combination with the weather conditions affected the cremation settings.

## Conclusion

This preliminary study highlights the significant impact of seasons and weather conditions on cremation processes, as investigated through experimental archaeology and state-of-the-art analytical techniques on burned remains. The findings underscore the important role of day temperature and the state of fuelwood during the experiments in achieving maximum burning temperatures more quickly. Dry fuelwood and elevated day temperatures facilitate the burning process. However, monthly precipitation emerged as a critical factor, consistently reducing burning temperatures regardless of the fuelwood’s condition.

Higher burning temperatures were observed during autumn and spring, as evidenced by both recorded temperatures and infrared indices (IRSF to C/C), suggesting a higher-intensity burning processes during these seasons. Moreover, the results highlight that IRSF values are influenced not only by temperature but also by the duration of high-intensity burning.

Fragmentation of the burned skeletal remains was uniform across all experiments, with no significant differences observed. This consistency is likely attributed to the absence of freezing temperatures or rainy weather conditions during the experiments and the collection of skeletal remains after burning.

Furthermore, atmospheric pressure and monthly precipitation levels were found to significantly influence the isotopic (δ^13^C and δ^18^O) values of the burned bones indicating that carbon and oxygen isotope analysis on bioapatite can be used for investigating in which season cremation was performed. Comparisons with archaeological data from Belgium reveal potential seasonal patterns in past cremation practices. These results underline the complexity of understanding the season in which cremations were performed and underscore the need for expanded experimental research in various geographical areas with different altitudes and weather conditions to further investigate how the location in which burning was performed in combination with the weather conditions affected the cremation settings and therefore the past funerary practices.

## Supporting information

S1 File Description of the Euclidean distance method used to assign archaeological samples to seasonal reference groups, including statistical analysis and results. (XLSX)

S1 Table Contingency table with assigned seasonal values for archaeological observations. (DOCX)

S2 Table Euclidean distance result data table (Excel file). (XLSX)

S3 File Inclusivity in global research questionnaire. (DOCX)
