# Supplementary material for: The seasonality experiment: Investigating how seasons affect the burning conditions of cremations
Source: PLoS One. 2025 Jul 9;20(7):e0327478. doi: 10.1371/journal.pone.0327478 (PMC12240392; doi:10.1371/journal.pone.0327478)
Supplement: S1 Table — (DOCX) [file pone.0327478.s002.docx]

**The seasonality experiment: Investigating how seasons affect the burning conditions of cremations**

Elisavet Stamataki^1,2^, Guy De Mulder^3^, Rosalie Hermans^1^, Martine Vercauteren^2^, & Christophe Snoeck^1^

1 *Archaeology, Environmental Changes, and Geo-Chemistry Research Unit, Vrije Universiteit Brussel, Pleinlaan 2, 1050, Brussels, Belgium*.

2 *Research Unit: Anthropology and Human Genetics, Department of Biology of Organisms and Ecology, Université Libre de Bruxelles, CP192, Avenue F.D. Roosevelt 50, 1050 Brussels, Belgium.*

3 *Department of Archaeology, Ghent University, Sint-Pietersnieuwstraat 35, 9000 Ghent, Belgium.*

**Supplementary Information**

**S1. Euclidean distance**

δ^13^C and δ^18^O values from both the archaeological and reference datasets were z-score standardised by subtracting the mean and dividing by the standard deviation for each variable. For the reference dataset, mean δ^13^C and δ^18^O values were calculated for each season (Winter, Spring, Summer, Autumn), yielding seasonal centroids. Euclidean distances between the δ^13^C and δ^18^O values of each archaeological sample and the seasonal centroids were calculated. Each archaeological sample was assigned to the season with the smallest Euclidean distance. A Chi-square test of independence was performed to assess the relationship between archaeological period (Bronze Age/Early Iron Age [LBA/EIA] and Roman period) and season assignment. A Chi-square test of independence was conducted to examine the relationship between assigned season and chronological period. The result was statistically significant, χ²(3) = 81.52, p-value < 2.2e-16, indicating that the distribution of seasons differs between the LBA/EIA and Roman periods.

**Table 1**. Contingency table with assigned seasonal values for archaeological observations.

|  | LBA/EIA | Roman |
| --- | --- | --- |
| Autumn | *29* | *136* |
| Spring | *41* | *51* |
| Summer | *26* | *70* |
| Winter | *111* | *65* |

The table with the Euclidean distances between the δ^13^C and δ^18^O values of each archaeological sample and the seasonal centroids has been uploaded as a .csv file.
